# Lifestyle behaviors associated with the population-level double burden of malnutrition among middle school students in Saudi Arabia: a cross-sectional study

**DOI:** 10.3389/fnut.2026.1930134

**Published:** 2026-10-30

**Authors:** Salwa Ali Marzouk, Asmaa Mohamed Ali AlAbd, Omaima Mohamed Elalem, Nojoud Abdullah Alrashidi, Sara Farhan Alenizi, Amjad Fahmi Othman, Anas Mahmoud Balawi

**Affiliations:** 1 Maternal and Child Health, College of Nursing, University of Ha’il, Ha’il, Saudi Arabia; 2 Psychiatric and Mental Health Nursing, College of Nursing, University of Ha’il, Ha’il, Saudi Arabia; 3 Community Health Nursing, College of Nursing, University of Ha’il, Ha’il, Saudi Arabia; 4 Medical-Surgical Nursing, College of Nursing, University of Ha’il, Ha’il, Saudi Arabia

**Keywords:** Body Mass Index, dietary habits, double burden of malnutrition, lifestyle behaviors, physical activity, Saudi adolescents, screen-based eating

## Abstract

**Background:**

The coexistence of undernutrition and overweight/obesity, commonly referred to as the double burden of malnutrition, is an important public health concern among adolescents. This study assessed the population-level double burden of malnutrition among middle school students and examined its associations with selected lifestyle behaviors.

**Methods:**

A cross-sectional study was conducted among 268 middle school students recruited from four schools selected using stratified cluster sampling. Analyses incorporated sampling weights and the complex survey design. Data on dietary habits, physical activity, and screen-related behaviors were collected using a structured self-administered questionnaire. The research team obtained anthropometric measurements, including height and weight. Nutritional status was classified using the World Health Organization BMI-for-age reference. Statistical analyses incorporated sampling weights and the complex survey design, with associations examined using the Rao–Scott design-adjusted chi-square test.

**Results:**

Survey-weighted estimates showed that 49.8% of the represented population had an abnormal BMI-for-age classification: 25.3% were underweight and 24.5% were overweight or obese, indicating a population-level double burden of malnutrition. In the design-adjusted bivariate analyses, eating while using screens was the only behavior significantly associated with BMI category (*p* = 0.046). The weighted prevalence of overweight/obesity increased from 12.1% among students who never ate while using screens to 32.9% among those who always did so. Demographic characteristics, dietary behaviors, physical-activity frequency, screen-time duration, mobile-phone use, and eating while using a mobile phone were not statistically significant.

**Conclusion:**

Underweight and overweight/obesity coexisted at similar levels in the represented middle-school population. Eating while using screens was associated with BMI category in the design-adjusted bivariate analysis; however, the cross-sectional design and limited number of sampled schools require cautious interpretation. The findings support school-health strategies addressing both forms of malnutrition and promoting balanced dietary practices, regular physical activity, and reduced screen-related eating.

## 1 Introduction

The double burden of malnutrition (DBM) has emerged as a public health issue among teenagers globally, particularly in societies experiencing rapid changes in diets and lifestyles. The concept of the double burden of malnutrition (DBM) describes the coexistence of undernutrition with overweight/obesity within the same population; this can be detected at the level of individual, household or community (1).

The coexistence of underweight and overweight/obesity in the same society has become even more obvious in teenage populations especially in countries undergoing urbanization, dietary transitions and more sedentary lifestyles like Saudi Arabia (1, 2). Adolescence is a critical developmental period characterized by rapid physical, psychological, and social changes. During this stage, individuals establish dietary and lifestyle habits that often continue into adulthood and substantially influence future health. As adolescents gain greater autonomy in food selection and daily activities while being increasingly exposed to peer, family, and media influences, their nutritional status becomes more vulnerable to both healthy and unhealthy behavioral patterns (3).

Saudi Arabia has experienced significant socioeconomic development over the past decades, accompanied by major changes in dietary practices and daily lifestyles. Greater consumption of energy-dense convenience foods, declining levels of physical activity, and increased recreational screen time have become common among adolescents. These behavioral changes may contribute to both inadequate and excessive nutritional status, thereby increasing the risk of adverse health outcomes during adolescence and later life (4–6). Recent national data indicate that malnutrition remains a significant public health concern among Saudi adolescents. A national multicenter population-based study reported overweight and obesity prevalence of approximately 11.2 and 9.4%, respectively, among Saudi children and adolescents (7), while a separate nationally representative study of Saudi adolescents reported an underweight prevalence of nearly 15% (8). These figures demonstrate that both forms of malnutrition coexist within the Saudi adolescent population at a substantial scale, underscoring the magnitude of the double burden of malnutrition and the need to identify modifiable lifestyle factors associated with this public health challenge.

Several modifiable lifestyle behaviors have been identified as important determinants of adolescents’ nutritional status. Unhealthy eating patterns, including frequent consumption of fast foods and sugar-rich snacks, skipping breakfast, insufficient participation in physical activity, and eating meals while watching screens or using mobile devices, have all been associated with an increased likelihood of abnormal body weight among adolescents (9, 10). However, most existing studies in this population have examined these behaviors in relation to overweight/obesity alone, without situating them within a double burden framework that also accounts for underweight. Evidence examining these behaviors across the full spectrum of nutritional status among Saudi middle school students therefore remains limited. The present study addresses this gap by examining multiple lifestyle behaviors in relation to underweight, normal weight, and overweight/obesity within a single sample, providing context-specific evidence that may support school-based public health interventions targeting the full spectrum of malnutrition. Screen-related eating behaviors may encourage distracted eating practices and increased intake of calorie-dense foods, potentially reducing adolescents’ awareness of normal hunger and fullness cues (11).

Both undernutrition and overweight in adolescence have deleterious effects on physical and psychological health. Adolescents with poor nutritional status can suffer decreased growth, low self-confidence, social problems and an increased risk of developing chronic health problems in later life. The coexistence of underweight and overweight/obesity among teenagers has become an emerging public health problem in Saudi Arabia especially in the setting of rapid lifestyle and dietary changes. This pattern of malnutrition may contribute to future metabolic and cardiovascular health problems and impact adolescents’ general well-being and school performance (5, 12).

Because lifestyle behaviors are often established during adolescence and may persist into adulthood, identifying modifiable behavioral factors associated with nutritional status is essential for developing effective school-based prevention strategies. Adolescents spend a large proportion of their time in the school environment, and schools may provide an important setting for promoting healthy eating habits, physical activity, and other lifestyle-related behaviors. Therefore, school-based health initiatives and supportive nutrition policies may help encourage healthier practices among students (13, 14).

Although widely previous investigations in Saudi Arabia have focused primarily on obesity, dietary behaviors, and physical inactivity among adolescents, fewer studies have explored the population-level underweight and overweight/obesity within the framework of the double burden of malnutrition, particularly among middle school students. In addition, evidence from the Ha’il region remains limited despite the noticeable lifestyle and dietary changes experienced by adolescents in recent years and the combined influence on nutritional status. Exploring factors associated with different forms of malnutrition may help guide the development of more targeted school-based strategies to improve adolescents’ nutritional health.

Therefore, this study aimed to assess the population-level double burden of malnutrition among middle school students in Ha’il, Saudi Arabia, and to examine the associations between BMI category and dietary habits, physical activity, and screen-related behaviors. The findings are expected to provide evidence that supports comprehensive school health programs and public health policies aimed at enhancing nutritional health of adolescents.

## 2 Materials and methods

### 2.1 Study design and setting

A cross-sectional study was conducted from October to the end of December 2024, during the 2024–2025 academic year, across four institutions representing all geographic regions of Ha’il City in Saudi Arabia, including both private and public schools.

### 2.2 Study population

The target population comprised Saudi students aged between 9 and 15 years, enrolled in one of the participating middle schools. Students with chronic illnesses, physical disabilities, diet-related health conditions, or those on medically prescribed diets were excluded to minimize the influence of medical conditions that could affect nutritional status.

### 2.3 Sample size and sampling technique

The sample size was calculated using OpenEpi (15), based on estimated prevalence rates of obesity and underweight from previous studies (7, 8). The higher of the two prevalence estimates was used for the calculation, with a 95% confidence level, 80% power, and a 5% margin of error. These parameters resulted in an eligible sample size of 246 students. The sample size was increased to 268 students to account for potential non-response. The calculation was primarily intended to support prevalence estimation and comparisons across BMI categories.

The study used stratified cluster sampling of schools followed by recruitment of eligible students. The sampling frame comprised 16 middle schools and was stratified by geographic region (East/West) and school sector (governmental/private), producing four strata: East governmental (6 schools), East private (2), West private (2), and West governmental (6). One school was selected at random from each stratum. In the four selected schools, 750 students met the eligibility criteria; all eligible students available during data collection were invited, and 268 participated and were included in the analysis (82, 61, 55, and 70 from schools 1–4, respectively). Figure 1 summarizes the sampling frame and participant flow.

**Figure 1:**
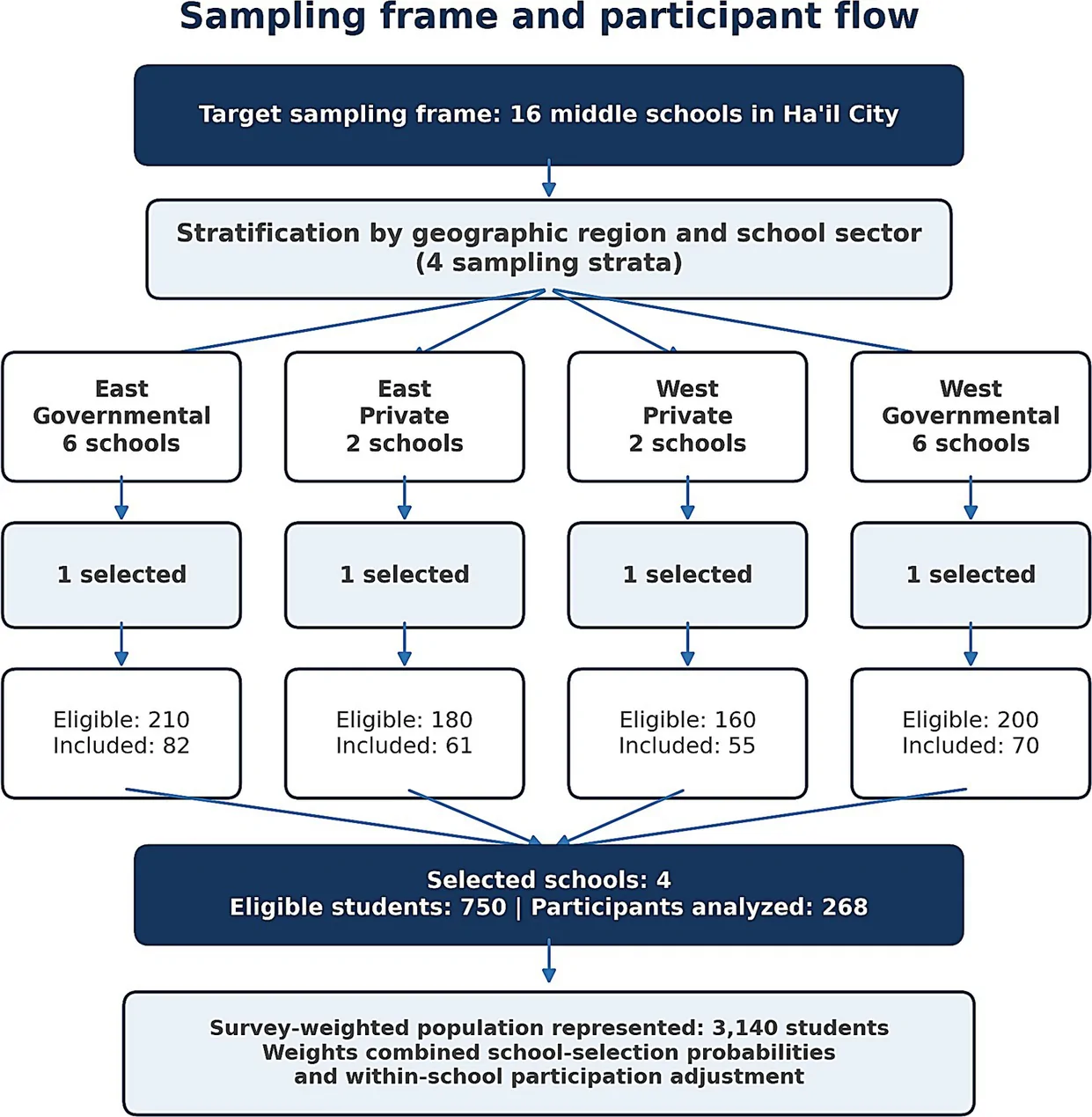
Sampling frame, school selection, participant recruitment, and weighted population represented. One school was randomly selected from each stratum; all eligible students available during data collection were invited.

### 2.4 Study instrument

Data were collected using a structured self-administered questionnaire consisting of three sections. The instrument primarily included closed-ended questions to obtain information on participants’ demographic characteristics, lifestyle behaviors, and anthropometric measurements. Lifestyle-related items focused on habitual dietary practices, physical activity, and screen use, with responses reflecting participants’ usual daily or weekly behaviors.

#### 2.4.1 Section 1: personal data

This section collected basic participant information, including age, sex, and school grade.

#### 2.4.2 Section 2: lifestyle practices

Lifestyle information was obtained using individual items adapted from previously published adolescent lifestyle instruments and relevant guidance sources addressing dietary habits, physical activity, and screen-related behaviors (16–18). The adaptation involved condensing and rewording selected items to reduce respondent burden and improve their suitability for Saudi middle-school students without introducing constructs beyond those covered in the source materials. The instrument was intended to provide epidemiological information on separate lifestyle practices rather than to measure a single latent construct. As described in Section 2.5, the adapted items underwent forward-backward translation, expert content review, and pilot testing to assess relevance, comprehensibility, and feasibility. The wording was simplified without changing the intended behavioral meaning. The complete questionnaire, response categories, and analytical variable definitions are presented in Supplementary Table S1. Lifestyle variables were grouped into dietary, physical-activity, and screen-related domains.

##### 2.4.2.1 Dietary habits

Information was collected on meal frequency, breakfast consumption, and the intake of selected food and beverage items, including fruits, vegetables, sweets, sugar-sweetened beverages, and fast food. Participants reported their usual consumption during the previous 30 days, and regular intake was defined as consuming a given food item at least once per week.

##### 2.4.2.2 Physical activity

Physical activity was evaluated using two items addressing the weekly frequency of participation in moderate-to-vigorous physical activity and the types of activities performed during a typical week.

##### 2.4.2.3 Screen-related behaviors

Participants reported their average daily recreational screen time and daily mobile-phone use, both categorized as ≤2 h or >2 h per day. They also reported eating while using screens and eating while using a mobile phone, with response options of never, sometimes, and always. All three response categories were retained separately in the design-adjusted bivariate analyses.

#### 2.4.3 Section 3: anthropometric measurements

Anthropometric measurements were obtained in the classroom following standardized measurement procedures. Standing height was measured to the nearest 0.1 cm using a portable stadiometer, with participants barefoot, standing upright with their heels together and their head positioned in the Frankfort horizontal plane. Weight was assessed to the nearest 100 g utilizing a calibrated portable electronic scale situated on a level surface, while participants donned lightweight attire and standing upright, barefoot. Following the measurement of height and weight, Body Mass Index (BMI) was computed as weight in kilograms divided by height in meters squared (kg/m^2^). Participants were classified in accordance with the World Health Organization growth reference for children and adolescents aged 5–19 years through the utilization of age- and sex-specific BMI-for-age Z-scores (19). Nutritional status was classified as underweight (< −2 SD), normal weight (−2 SD to +1 SD), overweight (> + 1 SD), or obese (> + 2 SD). For statistical analysis, overweight and obese participants were combined into a single category. The WHO BMI-for-age classification was used for epidemiological assessment of nutritional status. The study did not assess clinical, metabolic, or functional obesity-related health impairments; therefore, overweight and obesity categories should be interpreted as BMI-based weight status classifications rather than clinical obesity.

### 2.5 Data collection procedure

The questionnaire was originally formulated in English and subsequently translated into Arabic. A bilingual expert executed a forward translation from English to Arabic, followed by a reverse translation into English to ensure precision. To ensure the validity of the questionnaire, it was reviewed by eight experts specializing in Public Health and Pediatrics before data collection.

Their recommendations were incorporated to improve the relevance, comprehensibility, and content coverage of the questionnaire. A pilot study with 20 middle-school students assessed clarity, readability, feasibility, and completion time; pilot participants were excluded from the main analysis. Internal consistency was examined separately for the dietary, physical-activity, and screen-related domains using Cronbach’s alpha and mean inter-item correlations. Because the items represented heterogeneous behavioral indicators rather than unidimensional scales, these analyses were treated as supplementary diagnostics. No total domain scores were calculated, and each behavioral item was analyzed separately. Domain-specific internal-consistency results are presented in Supplementary Table S2.

Permission to conduct the study was obtained from the administrations of all participating schools before data collection commenced. Ethical approval was granted by the Institutional Review Board of the University of Ha’il prior to study implementation. Data were collected by trained research staff over a three-month period during regular school hours. They first consulted class teachers, then introduced the study to students and administered the questionnaire. Participants complete the questionnaire anonymously and receive the same instructions. Students completed the questionnaire in 10–15 min, with one researcher available for clarification. The second researcher performed and recorded anthropometric measurements in the same setting.

### 2.6 Data management and statistical analysis

Data were analyzed using IBM SPSS Statistics version 26.0 (20). The complex sampling design was incorporated into all survey analyses using IBM SPSS Complex Samples procedures. Schools were treated as primary sampling units, and the combined sampling weights reflected the inverse probability of school selection and the within-school participation adjustment. The original sampling design comprised four selection strata defined by geographical region and school sector, with one school selected from each stratum. For variance estimation, the governmental and private school strata were collapsed within each geographical region, producing two variance-estimation strata (East and West), with two school primary sampling units in each stratum. The original school-selection probabilities were retained in the calculation of the sampling weights. Categorical variables were summarized using unweighted counts and survey-weighted percentages. Associations between BMI category and categorical variables were evaluated using the Rao–Scott design-adjusted chi-square test. Survey-weighted estimates, design-adjusted standard errors, 95% confidence intervals, and *p*-values were reported, where estimable. Design effects were estimated for underweight and overweight/obesity.

A survey-weighted multinomial logistic regression model, with normal BMI as the reference outcome category, was initially explored. Candidate predictors were entered simultaneously using the Enter method. The Enter method was selected to examine the prespecified candidate predictors simultaneously and to avoid automated, data-driven stepwise selection. However, because the sampling design included only four primary sampling units and yielded two design degrees of freedom, the design-based covariance matrix was singular and the model standard errors and hypothesis tests were unstable. Therefore, the multivariable model and its estimates were not retained in the final analysis. Statistical significance was set at *p* < 0.05. No substantial missing data were identified. Reporting followed the STROBE guidance for cross-sectional studies.

## 3 Results

### 3.1 Baseline characteristics of the students studied

Table 1 presents the unweighted and survey-weighted characteristics of the studied students. Over two-thirds of the participants were female and aged 14–15 years. The distribution of participants across academic levels was relatively balanced among three educational stages. After applying the sampling weights, 25.3% of the target population was underweight, 50.2% had normal weight, and 24.5% was overweight or obese.

**Table 1:** Unweighted and survey-weighted characteristics of the study participants.

Variable	Category	Unweighted		Weighted	
		No	%	No	%
Sex	Male	86	32.1	1018.8	32.4
	Female	182	67.9	2121.2	67.6
Age group	12–13 years	93	34.7	1055.0	33.6
	14–15 years	175	65.3	2085.0	66.4
Academic level	7th	99	36.9	1106.1	35.2
	8th	85	31.7	1019.7	32.5
	9th	84	31.3	1014.2	32.3
BMI category	Underweight	71	26.5	793.9	25.3
	Normal	132	49.3	1577.3	50.2
	Overweight/Obesity	65	24.3	768.7	24.5

### 3.2 Body Mass Index (BMI) among the students

Figure 2 presents the BMI-category distribution. The weighted estimates indicated that underweight and overweight/obesity each affected approximately one-quarter of the represented population, indicating a considerable double burden of malnutrition.

**Figure 2:**
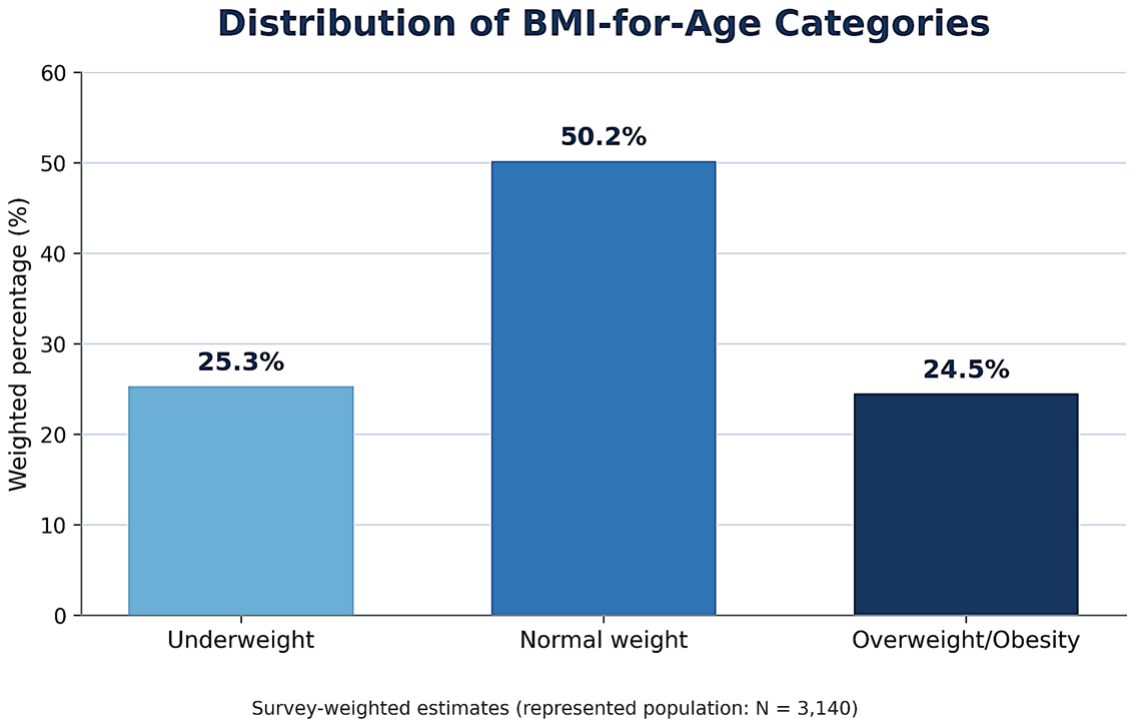
Distribution of BMI-for-age categories.

Table 2 shows the weighted distribution of BMI categories by sex, age group, and academic level. Underweight was descriptively more prevalent among males and younger adolescents, whereas overweight/obesity was descriptively more prevalent among females, older students, and ninth-grade students. However, after accounting for the complex sampling design, BMI category was not significantly associated with sex (*p* = 0.150), age group (*p* = 0.132), or academic level (*p* = 0.126).

**Table 2:** Design-adjusted associations between BMI category and demographic characteristics.

Variable	Category	Underweight		Normal weighted		Overweight/Obesity		Design-adjusted *p*-value
		No	%	No	%	No	%	
Sex	Male	32	35.1	40	47.8	14	17.1	0.150
	Female	39	20.6	92	51.4	51	28.0	
Age group	12–13 years	30	31.3	52	57.7	11	11.0	0.132
	14–15 years	41	22.2	80	46.5	54	31.3	
Academic level	7^th^	30	32.3	51	51.8	18	15.9	0.126
	8^th^	15	14.9	52	64.9	18	20.2	
	9^th^	26	29.9	29	34.5	29	34.5	

No. represents the unweighted count; percentages are survey weighted. *p*-values were obtained using the Rao–Scott design-adjusted chi-square test.

### 3.3 Students’ Body Mass Index (BMI) and its association with lifestyle behaviors

Table 3 presents the design-adjusted associations between BMI category and dietary behaviors. None of the examined dietary behaviors was statistically significantly associated with BMI category. Underweight prevalence varied across dietary categories without showing a consistent pattern. For example, underweight was more prevalent among students who regularly consumed breakfast than among those who did not (33.8% vs. 17.0%) and varied across fast-food frequency categories, from 24.3% among students reporting one fast-food meal per day to 32.7% among those reporting two meals and 10.3% among those reporting three or more. Underweight prevalence was similar among students who consumed sweets and those who did not (25.1% vs. 26.5%). In contrast, overweight/obesity was descriptively more prevalent among students who consumed sweets than among those who did not (32.0% vs. 13.4%) and among students who did not regularly consume breakfast compared with those who did (31.5% vs. 17.3%). Overweight/obesity also increased descriptively with fast-food frequency, from 19.8% among students reporting one fast-food meal per day to 39.7% among those reporting two meals and 61.6% among those reporting three or more. Nevertheless, the design-adjusted associations were not statistically significant for breakfast consumption (*p* = 0.078), sweet consumption (*p* = 0.103), or fast-food frequency (*p* = 0.110).

**Table 3:** Design-adjusted associations between BMI category and dietary behaviors.

Variable	Category	Underweight		Normal weighted		Overweight/Obesity		Design-adjusted *p*-value
		No	%	No	%	No	%	
No. of meals/day	<3	51	23.0	100	52.5	47	24.5	0.501
	≥3	20	31.3	32	44.3	18	24.4	
Eating breakfast regularly	Yes	48	33.8	63	49.0	24	17.3	0.078
	No	23	17.0	69	51.5	41	31.5	
Vegetables	Yes	48	23.6	95	57.0	33	19.4	0.203
	No	23	28.3	37	37.6	32	34.0	
Eating snacks/day	Yes	71	25.7	127	49.7	64	24.7	0.432
	No	0	0.0	5	87.3	1	12.7	
Fruits	Yes	55	26.3	92	50.2	44	23.5	0.462
	No	16	24.0	35	48.2	20	27.8	
Potato chips	Yes	55	26.6	95	46.6	52	26.8	0.229
	No	16	22.7	32	59.5	12	17.8	
Juice	Yes	54	26.0	98	52.0	41	22.0	0.213
	No	17	24.7	29	43.4	23	31.9	
Sweets	Yes	41	25.1	70	42.8	49	32.0	0.103
	No	30	26.5	57	60.2	15	13.4	
Milk	Yes	36	22.6	64	53.8	30	23.6	0.497
	No	35	28.4	63	45.9	34	25.6	
Drinking beverages	Yes	61	24.8	117	50.3	60	24.9	0.691
	No	10	29.1	15	49.8	5	21.1	
Beverage number/day	One	41	23.8	88	58.0	26	18.2	0.117
	Two	10	25.3	20	42.8	18	31.9	
	Three or more	10	28.8	9	24.2	16	47.0	
Eating fast food	Yes	38	23.7	70	46.3	47	30.0	0.181
	No	33	27.4	62	55.5	18	17.0	
Fast-food meals/day	One	26	24.3	55	55.8	23	19.8	0.110
	Two	9	32.7	9	27.7	11	39.7	
	Three or more	3	10.3	6	28.1	13	61.6	

No. represents the unweighted count; percentages are survey weighted. *p*-values were obtained using the Rao–Scott design-adjusted chi-square test.

Table 4 presents the design-adjusted associations of BMI category with physical activity and screen-related behaviors. Eating while using screens was the only behavior significantly associated with BMI category (*p* = 0.046). Underweight prevalence did not show a consistent pattern across the response categories, accounting for 21.8% among students who never ate while using screens, 28.0% among those who sometimes did so, and 22.6% among those who always did so. In contrast, overweight/obesity increased progressively from 12.1% among students who never ate while using screens to 26.5% among those who sometimes did so and 32.9% among those who always did so. Physical-activity frequency showed a possible but non-significant association with BMI category (*p* = 0.099): underweight was most prevalent among students reporting daily physical activity (33.2%), whereas overweight/obesity increased from 12.2% among students reporting daily activity to 40.0% among those reporting activity once weekly. For eating while using a mobile phone, underweight prevalence was relatively similar among students who never or sometimes did so (27.9 and 28.4%, respectively) and was lower among those who always did so (21.1%). In contrast, overweight/obesity increased descriptively from 7.4% among students who never ate while using a mobile phone to 19.2% among those who sometimes did so and 36.8% among those who always did so. However, this association was not statistically significant (*p* = 0.148). Screen-time duration (*p* = 0.371) and mobile-phone use duration (*p* = 0.755) were also not statistically significantly associated with BMI category.

**Table 4:** Design-adjusted associations between BMI category, physical activity, and screen-related behaviors.

Variable	Category	Underweight		Normal weight		Overweight/Obesity		Design-adjusted *p*-value
		No	%	No	%	No	%	
Physical activity	Daily	30	33.2	49	54.7	13	12.2	0.099
	Twice a week	19	18.6	51	58.7	21	22.8	
	Once a week	22	23.5	32	36.5	31	40.0	
Screen time/day	≤2 h	30	22.4	61	47.1	38	30.5	0.371
	>2 h	41	27.7	71	52.9	27	19.3	
Eating while using screens	No	16	21.8	39	66.1	7	12.1	0.046*
	Sometimes	42	28.0	66	45.5	37	26.5	
	Always	13	22.6	27	44.5	21	32.9	
Mobile use/day	≤2 h	38	26.9	66	50.1	34	23.0	0.755
	>2 h	33	23.5	66	50.4	31	26.2	
Eating while using mobile	No	13	27.9	28	64.7	3	7.4	0.148
	Sometimes	31	28.4	55	52.4	21	19.2	
	Always	27	21.1	49	42.1	41	36.8	

No. represents the unweighted count; percentages are survey weighted. *p*-values were obtained using the Rao–Scott design-adjusted chi-square test. Bold values indicate statistical significance (*p* < 0.05). *indicates statistical significance (*p* < 0.05).

### 3.4 Survey design effects

Table 5 presents the survey design effects for the primary nutritional-status outcomes. The design effect was 1.497 for underweight, indicating greater variance than expected under simple random sampling. For overweight/obesity, the design effect was 0.762, indicating lower variance under the specified complex sampling design. These estimates should be interpreted cautiously because only four school primary sampling units were included.

**Table 5:** Survey design effects for the primary nutritional-status outcomes.

Outcome	Unweighted prevalence %	Weighted prevalence %	Survey SE	95% CI	Survey DEFF
Underweight	26.5	25.3	0.031	11.9–38.7	1.497
Overweight/obesity	24.3	24.5	0.022	15.0–33.9	0.762

SE, standard error; CI, confidence interval; DEFF, design effect. Estimates should be interpreted cautiously because only four school primary sampling units were sampled.

## 4 Discussion

The present study assessed the population-level double burden of malnutrition and its associations with lifestyle behaviors among middle school students. Survey-weighted estimates showed that underweight and overweight/obesity coexisted at similar levels. In the design-adjusted bivariate analyses, eating while using screens was the only behavior significantly associated with BMI category. The multivariable model was not retained because the limited number of school primary sampling units produced unstable design-based covariance estimates. Accordingly, the findings should be interpreted as descriptive and bivariate associations rather than independent effects.

The study demonstrated that underweight and overweight/obesity coexist among middle-school students in Ha’il, Saudi Arabia. Approximately one-quarter of the represented population was underweight, whereas a similar proportion was classified as overweight or obese, indicating a population-level double burden of malnutrition. This pattern is consistent with the nutritional transition observed in Saudi Arabia, where undernutrition coexists with greater exposure to energy-dense diets, sedentary lifestyles, and prolonged recreational screen use. Within this context, nutritional deficiencies and excess body weight may occur in different subgroups alongside differing lifestyle and environmental circumstances. Similar patterns have been reported in Saudi Arabia and other middle-income settings undergoing social and nutritional change (1, 21–24). Collectively, the findings support comprehensive public-health strategies addressing both undernutrition and excess weight rather than focusing exclusively on obesity prevention.

After accounting for the survey design, none of the examined dietary behaviors was statistically associated with BMI category. The most noticeable descriptive differences involved breakfast consumption, sweet consumption, and fast-food frequency. Regular breakfast consumption has been associated with more regular appetite patterns, lower reported food intake later in the day, and better overall dietary quality. In the present study, overweight/obesity was descriptively more common among students who skipped breakfast, consumed sweets, and reported more frequent fast-food meals, whereas underweight showed no consistent pattern across these behaviors. These patterns may reflect differences in meal regularity and exposure to energy-dense foods, while underweight may be associated with unmeasured factors such as total dietary adequacy, portion size, restrictive eating, food insecurity, health conditions, or growth-related requirements. Similar associations between breakfast skipping, energy-dense dietary practices, frequent fast-food intake, and excess body weight have been reported among adolescents (9, 22, 24). However, the associations in the present study were not statistically significant and should be interpreted as descriptive patterns rather than independent associations or causal relationships.

Physical-activity frequency showed a possible but non-significant association with BMI category in the design-adjusted analysis (*p* = 0.099). Underweight was descriptively more common among students reporting daily physical activity, whereas overweight/obesity was more common among those reporting physical activity once weekly. This pattern should not be interpreted as an adverse effect of physical activity, as it may reflect differences in energy intake, growth, health status, or other unmeasured factors. The assessment of physical activity by frequency alone, without measuring duration or intensity, may also have limited the analysis. Regular physical activity nevertheless remains important for adolescent health and is supported by Saudi and international evidence (5, 10, 25).

Eating while using screens was the only screen-related behavior significantly associated with BMI category in the design-adjusted bivariate analysis. Underweight showed no consistent pattern across the frequency categories of eating while using screens, whereas overweight/obesity became descriptively more common as the frequency of this behavior increased. A similar descriptive pattern was observed for eating while using a mobile phone: underweight was relatively similar among students who never or sometimes engaged in this behavior and was lower among those who always did so, while overweight/obesity was progressively more common across the never, sometimes, and always categories. However, the association between eating while using a mobile phone and BMI category was not statistically significant. Previous studies have associated screen-related eating with lower awareness of satiety cues, distracted eating, and greater consumption of energy-dense foods (11, 14, 26). Prolonged screen use has also been associated with more sedentary behavior and fewer opportunities for physical activity. The present findings may suggest that eating in conjunction with screen use is more relevant to differences in weight status than screen duration alone. These interpretations remain tentative because meal size, food type, and the duration and context of screen use during eating were not assessed.

BMI category was not significantly associated with sex, age group, or academic level after accounting for the complex sampling design. Nevertheless, underweight was descriptively more prevalent among male and younger students, whereas overweight/obesity was more prevalent among female, older, and ninth-grade students. These descriptive patterns may be associated with biological and pubertal differences, activity patterns, academic demands, dietary practices, and body-image concerns; however, these explanations were not directly tested. Similar age- and sex-related patterns have been reported among Saudi adolescents (7, 12, 23). The descriptively higher prevalence of overweight/obesity among older adolescents may also be associated with age-related differences in physical activity and lifestyle; however, the association with age was not statistically significant, and the cross-sectional design does not establish temporal direction (27).

The exploratory survey-weighted multivariable model could not provide stable inferential estimates because the design included only four school primary sampling units and two design degrees of freedom. Therefore, no factor was interpreted as an independent predictor of underweight or overweight/obesity. This analytical limitation reinforces the need to interpret the observed patterns cautiously and to confirm them in studies sampling a larger number of schools.

The absence of statistically significant lifestyle correlates of underweight in the design-adjusted bivariate analyses does not mean that underweight has no modifiable correlates. Studies from Ethiopia and Guatemala have linked adolescent thinness to household and environmental circumstances, including family size, access to safe water and sanitation, and other demographic characteristics (28, 29). Psychological and social circumstances may also matter. Body-image concerns, restrictive eating, psychological distress, reduced appetite, and family or peer influences may be associated with differences in food intake during adolescence. Economic conditions, including household food insecurity, limited financial resources, and access to nutritious foods, may likewise be associated with nutritional status. Regular physical activity has also been inversely associated with underweight in another population (30), showing that patterns are not necessarily consistent across settings. We did not measure these psychological, social, economic, or household factors as primary determinants. They should therefore be regarded as plausible contextual explanations rather than explanations established by our data. Future studies should directly assess dietary adequacy, psychological well-being, household food security, socioeconomic circumstances, health conditions, and pubertal development when examining adolescent underweight.

The revised design-adjusted analyses did not identify a statistically significant dietary correlate of BMI category. Although overweight/obesity increased descriptively with fast-food frequency and sweet consumption, these patterns did not meet the conventional significance threshold. Accordingly, they should be treated as descriptive observations requiring confirmation in larger studies.

No single characteristic fully described the pattern of overweight/obesity observed in this study. Although the cross-sectional results cannot show that changing any behavior would alter BMI, schools remain a practical setting in which to address several aspects of adolescent health together. Programs could combine nutrition education, opportunities for physical activity, guidance on screen-related eating, and family involvement. This broader approach is consistent with international obesity-prevention guidance and school-health research (6, 13, 22).

The findings have public health implications for adolescent nutrition in Saudi Arabia. The coexistence of underweight and overweight/obesity supports comprehensive school-health strategies rather than an exclusive focus on obesity. Screen-related eating may be a useful topic for school nutrition education, while underweight assessment should also consider food security, total energy intake, health conditions, psychosocial factors, and pubertal development that were not captured in this study.

## 5 Strengths and limitations

One strength of the current study is that it investigated both underweight and overweight/obesity within the same group of adolescents, providing a broader view of malnutrition patterns among middle school students in the Ha’il region. In addition, anthropometric measurements were obtained directly using standardized techniques, avoiding reliance on self-reported height and weight. The study also examined dietary habits, physical activity, and screen-related behaviors, allowing several relevant lifestyle indicators to be considered within the same study.

Several limitations should be acknowledged. The cross-sectional design does not establish whether the measured behaviors preceded or followed the observed BMI categories; therefore, no causal inference can be made. Lifestyle behaviors were self-reported and may have been affected by recall or reporting bias. Moreover, the questionnaire captured selected behavioral indicators but did not assess the full complexity of dietary intake, physical activity, or screen-related behavior.

The study included only four school primary sampling units. Although the complex sampling design, selection weights, and clustering of students within schools were incorporated into the analyses, the resulting two design degrees of freedom limited the precision of the design-adjusted estimates. The small number of school clusters also prevented stable estimation of a survey-weighted multivariable multinomial regression model. Accordingly, the findings should be interpreted primarily as survey-weighted prevalence estimates and design-adjusted bivariate associations rather than independent associations or predictors. Although the weights incorporated school-selection probabilities and a within-school participation adjustment, student recruitment depended on availability and participation rather than random selection. Weighted estimates therefore assume that participants were representative of eligible students within each selected school; residual selection bias remains possible, and generalization beyond the defined sampling frame should be cautious.

Finally, several potentially relevant contextual variables were not collected, including socioeconomic status, parental education, household income, family lifestyle characteristics, sleep duration, pubertal development, total dietary energy intake, and psychological well-being. These unmeasured factors may have contributed to the observed differences across BMI categories and should be examined in future studies.

Furthermore, micronutrient status was not assessed using biochemical biomarkers, limiting our ability to identify micronutrient deficiencies or hidden hunger, which may occur even among adolescents with normal BMI or overweight/obesity. Consequently, the study may not fully capture the broader spectrum of malnutrition beyond BMI-based nutritional status.

## 6 Conclusion and recommendations

This study identified a survey-weighted coexistence of underweight (25.3%) and overweight/obesity (24.5%) among the represented middle-school population in Ha’il. Eating while using screens was the only behavior significantly associated with BMI category in the design-adjusted bivariate analyses. Because the multivariable model could not be estimated reliably with only four school primary sampling units, no factor was interpreted as an independent predictor.

School health strategies should address both forms of malnutrition through routine nutritional screening, balanced dietary guidance, opportunities for physical activity, and education on screen-related eating. Interventions focused on underweight should additionally assess dietary adequacy and relevant household, developmental, and health factors.

Coordinated involvement of schools, families, and healthcare providers is relevant to promoting healthy lifestyle practices during adolescence. Routine nutritional screening and assessment of lifestyle behaviors within school-health services may assist in identifying students at nutritional risk. Future multicenter longitudinal studies are needed to clarify temporal relationships between lifestyle behaviors and nutritional status and to identify additional factors associated with the double burden of malnutrition among Saudi adolescents.

## Data Availability

The raw data supporting the conclusions of this article will be made available by the authors, without undue reservation.
