# Supplementary material for: Lifestyle behaviors associated with the population-level double burden of malnutrition among middle school students in Saudi Arabia: a cross-sectional study
Source: Front Nutr. 2026 Oct 30;13:1930134. doi: 10.3389/fnut.2026.1930134 (PMC13759813; doi:10.3389/fnut.2026.1930134)
Supplement: Supplementary file 1 [file Table_1.pdf]

Supplementary Table S1. Lifestyle-behavior questionnaire items, response options, and categorization used in the analysis

Domain	Variable	Questionnaire item	Response options	Categorization used in the analysis
**Dietary habits**	Breakfast consumption	Do you usually consume breakfast daily?	Yes / No	Yes vs. No
	Meal frequency	How many meals do you usually consume per day?	1–2 / 3 / ≥4 meals	<3 vs. ≥3 meals/day
	Vegetable consumption	During the past 30 days, did you regularly consume vegetables? (Regular intake: ≥1 time/week)	Yes / No	Yes vs. No
	Snack consumption	During the past 30 days, did you consume snacks during the day?	Yes / No	Yes vs. No
	Fruit consumption	During the past 30 days, did you regularly consume fruits? (Regular intake: ≥1 time/week)	Yes / No	Yes vs. No
	Milk consumption	During the past 30 days, did you regularly consume milk?	Yes / No	Yes vs. No
	Potato-chip consumption	During the past 30 days, did you regularly consume potato chips? (Regular intake: ≥1 time/week)	Yes / No	Yes vs. No
	Sweet/chocolate consumption	During the past 30 days, did you regularly consume sweets or chocolate? (Regular intake: ≥1 time/week)	Yes / No	Yes vs. No
	Juice consumption	During the past 30 days, did you regularly consume juice? (Regular intake: ≥1 time/week)	Yes / No	Yes vs. No
	Sugar-sweetened beverage consumption	During the past 30 days, did you consume soft drinks or other sweetened beverages?	Yes / No	Yes vs. No
	Beverage frequency among consumers	How many sweetened beverages did you usually consume per day?	One / Two / Three or more	1 / 2 / ≥3 per day
	Fast-food consumption	During the past 30 days, did you consume fast food?	Yes / No	Yes vs. No

	Fast-food frequency among consumers	How many fast-food meals did you usually consume per day?	One / Two / Three or more	1 / 2 / ≥3 meals/day
**Physical activity**	Weekly activity frequency	How frequently did you engage in physical activity?	Daily / Twice weekly / Once weekly	Three categories retained
	Type of physical activity (multiple response)	Which types of physical activity did you usually perform?	Football / Swimming / Karate / Gymnastics / Cycling / Running or walking	Each activity coded separately as Yes/No
**Screen-related behaviors**	Daily screen time	How many hours per day did you spend watching television or using screens?	≤2 hours / >2 hours	≤2 vs. >2 hours/day
	Eating while using screens	How often did you eat while watching television or using screens?	Never / Sometimes / Always	Three categories retained
	Daily mobile-phone use	How many hours per day did you usually spend using a mobile phone?	≤2 hours / >2 hours	≤2 vs. >2 hours/day
	Eating while using a mobile phone	How often did you eat while using a mobile phone?	Never / Sometimes / Always	Three categories retained

Notes: The domains are content-based groupings rather than psychometric subscales; therefore, no composite lifestyle score was calculated. Conditional beverage- and fast-food-frequency variables were analyzed only among students reporting the corresponding consumption. The physical-activity-type item was multiple-response, and each activity was coded separately. Exact item wording should be cross-checked against the original questionnaire before submission.
