# Supplementary material for: Lifestyle behaviors associated with the population-level double burden of malnutrition among middle school students in Saudi Arabia: a cross-sectional study
Source: Front Nutr. 2026 Oct 30;13:1930134. doi: 10.3389/fnut.2026.1930134 (PMC13759813; doi:10.3389/fnut.2026.1930134)
Supplement: Supplementary file 2 [file Table_2.pdf]

Supplementary Table S2. Internal-consistency assessment of the behavioral questionnaire sections

Section/domain	Analysis	No. of indicators	Complete cases	Cronbach's α	Standardized α	Mean inter-item r	Interpretation
Dietary behavior indicators	Main section	11	262	0.383	0.418	0.061	Low internal consistency; analyze indicators separately, not as a total score.
Physical activity indicators	Main section	7[a]	268	-0.481	-0.559	-0.054	Negative coefficient, suggesting that the indicators may not form a unidimensional reflective scale.
Screen-related behavior indicators	Main section	4	268	0.152	0.136	0.038	Low internal consistency; analyze indicators separately, not as a total score.
Health-promoting dietary indicators	Sensitivity split	5	262	0.313	0.297	0.078	Low internal consistency; analyze indicators separately, not as a total score.
Less-healthful dietary indicators	Sensitivity split	5	262	0.415	0.421	0.127	Low internal consistency; analyze indicators separately, not as a total score.

[a] The physical-activity section comprised a frequency item and multiple-response indicators of activity type. Because these indicators represented heterogeneous behaviors rather than interchangeable items measuring a single construct, Cronbach's alpha is presented only as a supplementary diagnostic. No composite physical-activity score was calculated, and the indicators were analyzed separately.

Internal-consistency assessment

Internal consistency was low for the dietary and screen-related behavioral sections and negative for the physical-activity section. These coefficients indicate that the items do not form unidimensional reflective scales. Therefore, individual behaviors were analyzed separately and were not combined into composite dietary, physical-activity, screen-behavior, or overall lifestyle scores.
